# Ultrasound-guided foam sclerotherapy for chronic venous disease with ulcer. A prospective multiple outcome cohort study^*^

**DOI:** 10.1590/1677-5449.180108

**Published:** 2020-03-10

**Authors:** Guilherme Camargo Gonçalves de Abreu, Otacílio de Camargo, Márcia Fayad Marcondes de Abreu, José Luis Braga de Aquino

**Affiliations:** 1 Pontifícia Universidade Católica de Campinas – PUC-Campinas, Campinas, SP, Brasil.; 2 Hospital e Maternidade Celso Pierro, Serviço de Cirurgia Vascular e Endovascular, Campinas, SP, Brasil.

**Keywords:** varicose ulcer, leg ulcer, varicose veins, sclerotherapy, ultrasonography, quality of life, varizes, insuficiência venosa, úlcera varicosa, escleroterapia, ultrassonografia, qualidade de vida

## Abstract

**Background:**

Chronic Venous Disease (CVD) is the main cause of chronic leg ulcers. Varicose veins are the most frequent cause of venous leg ulcers (VLU). 50.9% of Brazilian women have varicose veins and ulcer prevalence is as high as 4%. Ultrasound-guided foam sclerotherapy (UGFS) is a low-cost treatment option for varicose veins.

**Objectives:**

To analyze UGFS outcomes in patients with VLU.

**Methods:**

Prospective consecutive single center cohort study. Patients with great saphenous vein (GSV) reflux and VLU were treated and followed-up for 180 days. The following were studied: quality of life (QoL), disease severity, healing, and elimination of GSV reflux. The Aberdeen questionnaire, a venous clinical severity score, and Duplex scanning (DS) results were analyzed.

**Results:**

22 patients aged 35 to 70 years were treated. There was improvement in quality of life, disease severity reduced, and ulcer diameter reduced (p < 0.001; ANOVA). 77.27% of VLU healed completely (95%CI: 59.76-94.78%). The dimensions of 20/22 VLU reduced (90.91%; 95%CI: 78.9-100%). GSV reflux was eliminated in 63.64% (95%CI: 43.54-83.74%). Men had greater QoL benefit and women had more complications. There were no severe complications. The VLU that had healed completely at the end of the study were smaller at baseline than those that did not completely heal. The GSV that were completely occluded at the end of the study were smaller at baseline than those that were not completely occluded (p < 0.05; Mann-Whitney).

**Conclusion:**

The results suggest that most patients benefited from UGFS.

## Resumo

**Contexto:**

A doença venosa crônica (DVC) é a principal causa de úlceras crônicas em membros inferiores. As varizes dos membros inferiores são a causa mais frequente de úlcera venosa (UV). No Brasil, 50,9% das mulheres têm varizes e a prevalência da úlcera chega a 4%. A escleroterapia ecoguiada com espuma (EEE) é uma alternativa de baixo custo para tratamento de varizes de membros inferiores.

**Objetivos:**

Analisar evolução de portadores de UV tratados com EEE.

**Métodos:**

Coorte prospectiva e consecutiva em um único serviço. Portadores de UV com veia safena magna (VSM) insuficiente foram acompanhados por 180 dias após EEE. Foram estudadas: qualidade de vida, gravidade da doença, cicatrização e eliminação do refluxo. Foram utilizados questionário Aberdeen, escore clínico venoso e ultrassom Doppler.

**Resultados:**

Foram tratados 22 pacientes com idade entre 35 a 70 anos. Houve melhora na qualidade de vida, redução da gravidade da doença, e redução dos diâmetros das úlceras (p < 0.001; ANOVA). Houve redução das dimensões em 90,91% das úlceras [intervalo de confiança de 95% (IC95%) 78,9-100%], e cicatrização completa em 77,27% (IC95% 59,76-94,78%). O refluxo foi eliminado em 63,64% (IC95% 43,54-83,74%) das VSM. Homens tiveram maior benefício em qualidade de vida, e mulheres apresentaram mais complicações. Não houve complicações graves. As UV completamente cicatrizadas e as VSM que apresentaram oclusão completa apresentavam dimensões inicialmente menores quando comparadas às das UV não completamente cicatrizadas e das VSM não completamente ocluídas (p < 0,05; Mann-Whitney).

**Conclusões:**

Os resultados sugerem que EEE foi benéfica para a maioria dos pacientes.

## INTRODUCTION

Chronic Venous Disease (CVD) is the main cause of lower limb chronic ulcers.^1^ Primary varicose veins with great saphenous vein (GSV) reflux is the most frequently identified condition in patients with venous ulcers (VU).^2^ It is estimated that 6% of patients with varicose veins will develop VU at some point in their lives.^3^ In Brazil, 50.9% of women and 37.9% of men have varicose veins and the prevalence of VU is as high as 3.6%.^4^ From 2009 to 2013, the Brazilian government spent about 125 million US Dollars on treatment and social security benefits for patients because of varicose veins and its complications.^5^^,^ 6 Specialized services improve rates of VU healing and reduce ulcer prevalence.^7^^,^ 8 Varicose vein surgery is considered economically advantageous for reducing VU recurrence.^9^

Treatment of primary CVD through compression therapy does not solve venous reflux. Compression is associated with healing of 65% of ulcers within 24 weeks, but up to 70% of the patients have VU recurrence when they end treatment.^10^^-^ 14 Surgery improves quality of life in varicose veins patients.^15^ Resection of insufficient GSVs reduces recurrence of VU.^12^^,^ 16 ^,^ 17 Foam sclerotherapy can provoke occlusion of over 80% of GSVs treated.^18^^,^ 19 Brittenden et al. reported 54% occlusion of veins in a large randomized trial.^20^ Divergent results are probably a reflection of heterogeneous anatomical and clinical characteristics in the populations studied. Furthermore, there is not merely one uniform foam sclerotherapy method. Controlled trials identify lower reflux cessation rates and higher reintervention rates after sclerotherapy when compared with surgery and thermoablation.^20^^,^ 21 Controlled trials also demonstrate that frequencies of adverse effects are similar after surgery and thermoablation.^21^^,^ 22 Notwithstanding the inferior result in terms of reflux cessation, patients treated with sclerotherapy present less pain, better quality of life, and a faster return to daily activities than patients treated surgically.^19^^,^ 21 ^,^ 23 Cost analyses favor sclerotherapy over other methods.^21^^,^ 24

## OBJECTIVES

To investigate quality of life, ulcer healing and venous disease severity after foam sclerotherapy treatment and investigate factors related to main outcomes.

## METHODS

**Figure 1:**
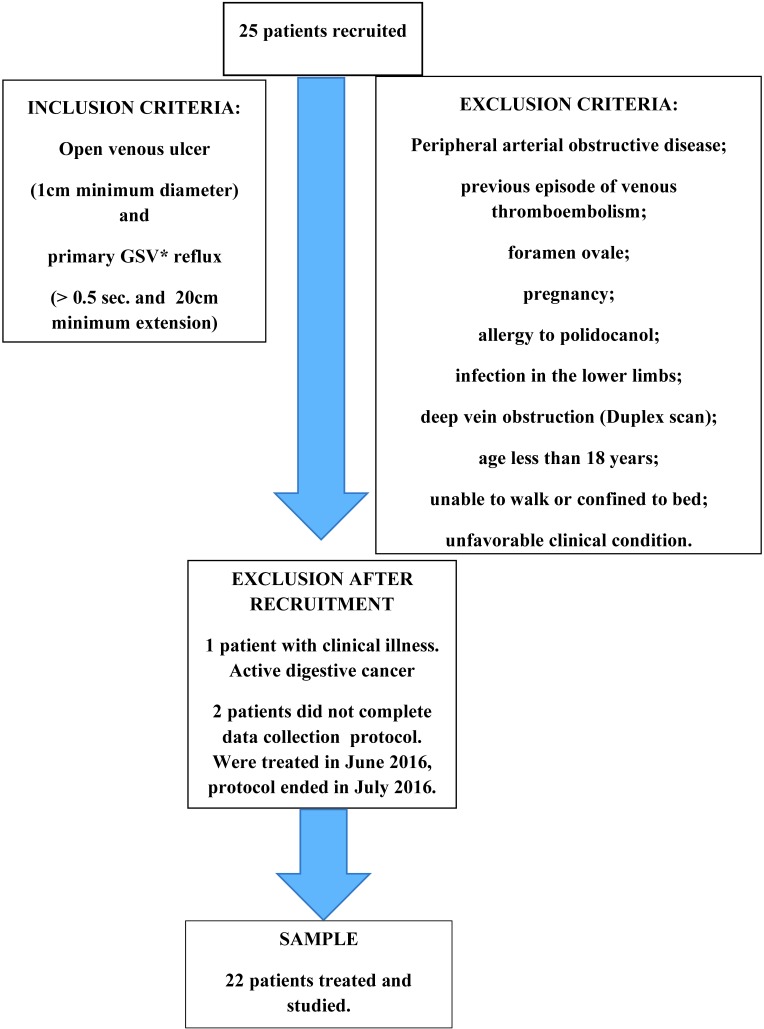
Sample flowchart. *Great saphenous vein.

Prospective and consecutive single center cohort with systematic follow-up and data collection protocol. All patients with non-healing venous ulcers and primary reflux of the GSV treated at the Hospital run by the Pontifícia Universidade Católica de Campinas (PUC-Campinas), Campinas, SP, Brazil, from June 2015 to June 2016. The study was approved by the ethics committee at the Pontifícia Universidade Católica de Campinas (PUC-Campinas). Only patients who were able to understand the risks and benefits and agreed to take part were included. We defined pathological reflux as duration greater than half a second and extension exceeding 20cm on a great saphenous vein Duplex scan (DS) performed standing up.^25^ MedisonSonoace Pico and Siemens Acuson X300 PE equipment were used. A certificated researcher performed all examinations. We defined venous ulcer as an open wound of at least 1cm diameter on the skin of the leg or foot, in areas with venous hypertension. Clinical examination was confirmed by DS^26^ (Figure 1).

The exclusion criteria were:

- Peripheral arterial obstructive disease;
- Venous thromboembolism
- Foramen ovale;
- Pregnancy;
- Allergy to polidocanol;
- Infection in the lower limbs;
- Deep vein obstruction (by Duplex scan);
- Age less than 18 years;
- Patients who were unable to walk or were confined to bed;
- Patients with unfavorable clinical conditions.

**Figure 2:**
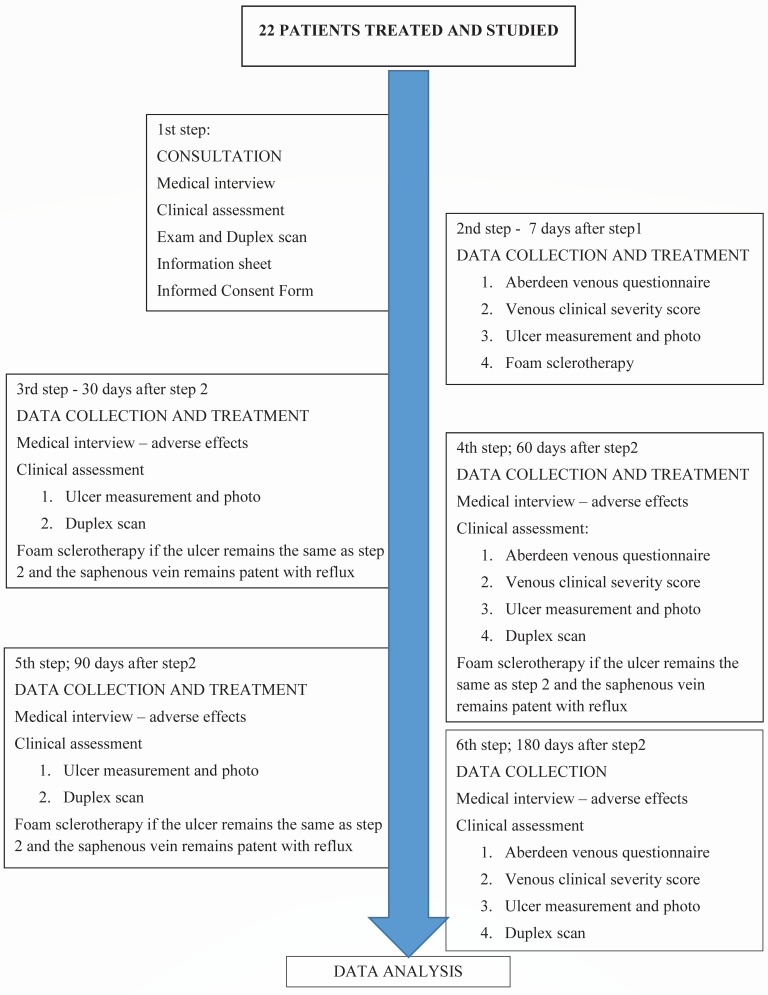
Protocol flowchart.

The following were evaluated: quality of life, venous disease severity, ulcer size, and venous status at baseline before treatment and at 60 days and 180 days after treatment. Versions of the Aberdeen questionnaire for venous disease (AQ) and the venous clinical severity score (VCSS) translated and validated for our language were used.^27^^-^ 29 Ulcer healing was evaluated by the largest ulcer diameter. Venous status was characterized by DS. Clinical, anthropometric, anatomical, and social data were collected (Figure 2).

In each session, 10 ml of foam was injected straight into the GSV with a single puncture. Puncture and foam progression were monitored by DS. Foam was produced by mixing 8ml of room air with 2ml of 3% polidocanol solution (Victalab).

Elastic bandages were applied after injections. Patients were instructed to keep the compression bandages on for 24 hours and wear 20 to 30 mmHg thigh-high compression stockings after removing the bandages. Patients were told to maintain their daily habits. Up to four treatment sessions were performed if there was persistent great saphenous vein reflux and if ulcer dimensions remained unchanged.

### Data analysis

Quality of life (assessed by AQ), severity score, and ulcer diameters evaluated at pre-treatment baseline and 60 days and 180 days after treatment were compared using repeated measures ANOVA on ranks (non-parametric method). Interactions between clinical variables and outcomes were also evaluated by ANOVA on ranks. Results for QoL, VCCS, and VU healing were classified as: improvement, no improvement, or worsening. Frequencies were described at each follow-up point, 60 and 180 days after treatment. Results of the great saphenous vein treatment were classified as complete or not complete occlusion and presence or absence of residual reflux. Results classified by categories were expressed as frequencies for each follow-up point. Patients were grouped according to outcome (VU healing, occlusion and cessation of reflux in the GSV, and occurrence of adverse effects). Continuous variables were compared using the Mann-Whitney test and categorical variables were compared using Fischer's test. A 5% statistical significance level was adopted. The Statistical Analysis System for Windows 9.4 (SAS Institute Inc., Cary, NC, USA) and Minitab 16 were used for statistical analysis.

## RESULTS

Twenty-two patients were treated consecutively. There were no losses. There were 42 treatments (1.9 +/- 0.9 application per patient). Most patients were obese or overweight, BMI varied between 23 and 45 (30 +/- 1), and only 3 patients (14%) had normal weight (BMI between 18.5 and 24.9). Ten patients (45%) did not have comorbidities whereas twelve (55%) patients did (Tables 1 and 2). Thirteen patients (59%) had no adverse effects. There were eleven adverse effects in nine patients, all of them female. Among the women, 60% had at least one adverse effect and 20% exhibited staining along the path of the vein treated. All adverse effects were mild and treated on an outpatient basis. There was a higher proportion of women in the group that presented at least one adverse effect (P = 0.017; Fisher). Other anatomical, clinical and social variables did not differ between these groups (with or without adverse effects) (Table 3).

**Table 1:** Distribution of number of administrations per patient.

**Patient (%)**	**Number of administrations**	**Total administrations**
8 (36.4%)	1	8
10(45.5%)	2	20
2 (9.1%)	3	6
2 (9.1%)	4	8
22 (100%)		42

**Table 2:** Measures of position (average and median) and dispersion (standard deviation, minimum and maximum) of numerical variables.

**Variable**	**Mean**	**Sd** ^§§^	**Min**	**Median**	**Max**
Administrations (n)	1.91	0.92	1.00	2.00	4.00
Age (years)	56.05	10.46	35.00	58.50	70.00
Schooling (years)	6.36	4.39	0.00	6.00	15.00
Baseline AQ^*^	47.05	12.17	21.70	45.00	74.20
Day 60 AQ*	23.96	10.29	6.30	21.20	48.20
Day 180 AQ*	16.99	9.58	0.00	16.40	37.00
Baseline VCSS^†^	18.64	3.03	14.00	19.00	24.00
Day 60 VCSS†	10.09	4.76	3.00	9.00	20.00
Day 180 VCSS†	6.73	3.61	1.00	6.00	16.00
VU^‡^ baseline diameter (cm)	4.11	3.40	1.00	3.00	14.00
VU‡ day 60 diameter (cm)	1.15	2.52	0	0	8.0
VU‡ day 180 diameter (cm)	1.18	2.65	0	0	10.0
GSV^‡‡^ baseline diameter (mm)	12.00	3.62	5.50	11.05	21.00
BMI^§^ (kg/m^2^)	30.2	5.4	23.1	30.3	44.7

* Aberdeen venous questionnaire;

† Venous clinical severity score;

‡ Larger venous ulcer diameter;

‡‡ Great saphenous vein;

§ Body mass index;

§§ standard deviation

**Table 3:** Distribution of adverse effects (AE) per patient (n).

**Adverse Effect**	**n**	**Patient with AE (n)**
Phlebitis with pain	4	3*, 13*, 15*, 19* (4)
Phlebitis without pain	2	2*, 4* (2)
Skin staining	3	4*, 12*, 15* (3)
Local pain	1	22*
Local cellulitis	1	16* (1)
Total AE	11	2*, 3*, 4*, 12*, 13*, 15*, 16*, 19*,22* (9)

* Patient affected by adverse effect.

### Quality of life

Twenty-one patients (95.45%; 95%CI: 86.75-100%) exhibited QoL improvement (reduction in AQ) by day 60. On day 180, all 22 (100%) patients had improved QoL compared to baseline. Five patients (22.73%; 95%CI: 5.22-42.24%) had deterioration of QoL from day 60 to day 180. Overall, QoL improved over time (p <0.001; ANOVA). There was an interaction between the number of UGFS sessions and quality of life (p-value = 0.0253). The worse the QoL assessed on day 60, the more treatment sessions the patients needed (ρ = 0.5449, p-value = 0.0087, Spearman). There was an interaction between gender and QoL progress (p = 0.0309). There were no differences in men’s and women’s pre-treatment QoL (p-value = 0.9438; Mann-Whitney) or day 60 QoL (p = 0.1805; Mann-Whitney). On day 180, men’s QoL was better than women’s (p=0.0074; Mann-Whitney). Both genders exhibited quality of life improvement over time (p <0.001; ANOVA); however, women’s QoL did not change from day 60 to day 180 (p = 0.0884). Among men, there were improvements at each evaluation (p <0001 baseline to day 60, p-value <0.001 baseline to day 180, p = 0.0393 from day 60 to day 180). The variables body mass index (BMI), GSV reflux pattern, VU diameter, GSV diameter, reflux in other venous territories, age, comorbidities, side affected by VU, educational level, and occupation did not have any significant influence.

### Clinical disease severity

Twenty-one patients (95%; 95%CI: 86.75-100%) exhibited reduction in severity by day 60. All 22 (100%) patients exhibited reduction in severity by day 180. Three (14%; 95%CI: 0-27.98%) exhibited deterioration between the evaluations on day 60 and day 180. Overall, severity reduced over time (p <0.001; ANOVA). Patients who had larger ulcers (ρ = 0.4350, p-value = 0.0430), older patients (ρ=0.4323; p-value =0.0445), women (p-value =0.0357), and those on sick leave (p-value = 0.0143) had more severe disease. The variables BMI, GSV reflux pattern, GSV diameter, reflux in other venous territories, number of treatment sessions administered to patients, comorbidities, side affected by VU, and educational level did not have any significant influence on the course of clinical severity.

### Venous ulcer

At baseline, all patients had non healing VU. On day 60, 15/22 VU (68.18%; 95%CI: 48.72-87.64%) had healed completely and the remainder were all smaller. On day 180, 17/22 (77.27%; 95%CI: 59.76-94.78%) ulcers had healed completely. Between day 60 and day 180 assessments, 3/22 (13.64% 95%CI: 0-27.98%) VU increased in size. There was no recurrence or new VU over the period (Tables 4, 5, 6, and 7).

**Table 4:** Frequency, percentage of ulcer healing and Confidence interval.

**Ulcer healing**	**n**	**% (95%CI**†**)**
Day 60		
Complete	15	68.18 (48.72-87.64%)
Incomplete	07	31.82 (12.36-52.28%)
Failure	0	0
Day 180		
Complete	17	77.27 (59.75-94.79%)
Incomplete	03	13.64 (0-27.98%)
Failure	02	9.09 (0-21.10%)

† Confidence interval

**Table 5:** Influence of variables on reduction in ulcer diameter.

**Variable**	**Interaction**	**p-value**
BMI^*^	0.5877	0.2897
GSV^†^ reflux pattern	0.4524	0.3242
GSV† diameter	0.9529	0.8751
Reflux in perforating veins	0.1991	0.2858
Reflux in deep veins	0.3636	0.2322
Number of applications	0.6992	0.9772
Gender	0.1002	0.0801
Age (years)	0.2449	0.1497
Comorbidity	0.2292	0.2243
Side affected by ulcer	0.9873	0.6225
Schooling (years)	0.5385	0.5509
Occupation	0.3349	0.1305

ANOVA on ranks

* Body mass index;

† Great saphenous vein.

**Table 6:** Ulcer diameter (in centimeters) during follow-up.

**Time**	**n**	**Average**	**Sd** ^*^	**Min.**	**Median**	**Max**
Baseline	22	4.11	3.4	1.00	3.00	14.00
Day 60	22	1.15	2.52	0.00	0.00	8.00
Day 180	22	1.18	2.65	0.00	0.00	10.00

* Standard deviation.

**Table 7:** Comparison of baseline venous ulcer diameters (in centimeters) between groups with complete and incomplete healing.

**VU healing**	**n (%)**	**Mean**	**Sd** ^*^	**min**	**median**	**max**	**p-value**
Healing at day 60							
Complete	15 (68.18%)	2.93	1.58	1.00	3.00	5.00	0.0336
Incomplete	7 (31.82%)	6.86	4.88	1	5.00	14.00	
Healing at day 180							
Complete	17 (77.27%)	2.94	1.64	1.00	3.00	5.00	0.0115
Incomplete	5 (22.73%)	8.4	4.83	3.00	9.00	14.00	

Mann-Whitney test.

* Standard deviation.

VU diameters reduced over time (p <0.001; ANOVA); however, they did not vary significantly between day 60 and day 180 (p-value = 0.8903). The variables BMI, GSV reflux pattern, GSV diameter, reflux in other venous territories, age, comorbidities, side affected by VU, educational level, and occupation did not exhibit any significant influence. Ulcers that healed had smaller diameters at baseline than those that did not completely heal (p-value = 0.0336 on day 60 and p-value = 0.0115 on day 180; Mann-Whitney)

### GSV occlusion and reflux

Initially, all GSVs had reflux. On day 60, reflux had been eliminated in 15/22 GSVs (68.18%; 95%CI: 48.72-87.64%) and on day 180 in 14/22 GSVs (63.64%; 95%CI: 43.54-83.74%). Total occlusion of GSVs ranged from 10/22 (45.45%; 95%CI: 24.64-66.26%) on day 60 to 7/22 (31.82%; 95%CI: 12.36-51.28%) on day 180. The GSVs that were completely occluded at the end of the study were smaller at baseline than those that were not completely occluded on day 60 (p = 0.003 Mann-Whitney) and on day 180 (p = 0.01 Mann-Whitney). The variables number of sclerotherapy sessions, body mass index (BMI), age, educational level, gender, relationship with work, existence of comorbidities, pattern of venous reflux, side affected, and occurrence of adverse effects did not differ between groups. When the group in which there was residual reflux in the GSV was compared with the group in which reflux was eradicated on day 180, they did not differ in relation to any of the variables studied (Mann-Whitney test and / or Fisher's test) (Tables 8 and 9).

**Table 8:** Comparison of baseline Great saphenous vein diameter between groups with partial and total GSV occlusion 60 days after treatment.

	**Partial Occlusion**	**Total Occlusion**	**p-value**
Baseline diameter Mean Sd (n)	13.8 ± 3.0 (n=12)	9.6 ± 2.8 (n=10)	0.003
Baseline diameter Median (min – max)	13.7 (1.7-21)	10.5 (5.5-14.8)	

Mann-Whitney test; Diameters in millimeters.

**Table 9:** Comparison of the baseline Great saphenous vein diameter between groups with partial and total GSV occlusion 180 days after treatment.

	**Partial Occlusion**	**Total Occlusion**	**p-value**
Baseline diameter Mean Sd (n)	13.22± 3.2(n=15)	9.4 ± 3.3 (n=7)	0.01
Initial diameter Baseline (min – max)	12.4 (8.7-21)	10.7 (5.5-14.8)	

Mann-Whitney test; Diameters in millimeters.

## DISCUSSION

Our protocol allowed selection of individuals considered to be poor candidates for surgery and who would benefit from elimination of venous reflux. Consecutive selection according to inclusion and exclusion criteria allowed the sample to be clinically homogenous and also enabled exclusion of patients at high risk of complications. No sample size calculations were conducted prior to the study. This was because multiple outcomes would be studied that would have different frequencies and would need samples of different sizes. Considering a healing rate of 91.3%, as reported by Campos et al., our study would have needed 123 cases to attain a 95% confidence interval (CI).^22^ It is important to note that small sample sizes can lead to unreliable results. Our cohort study is a longitudinal research project that aims to establish a causal link between events. It does not enable efficacy to be determined. Efficay testing would require a comparative study with control group.

We studied patients who spontaneously sought treatment and there was no active screening for these patients. It is likely such patients were more symptomatic and had worse quality of life and so presented great improvement in this regard.

A non-parametric method was used to compare VU diameter, VCSS, and AQ results. Non-parametric methods are best suited to avoid errors in small patient samples with data that are not normally distributed.

The predominance of overweight women (68%), obesity (86%), comorbidities (55%), low educational level (77% did not complete high school), and informal employment is similar to the population studied to validate the Brazilian version of the Aberdeen questionnaire.^27^^,^ 28

### Adverse effects

Wright et al.^19^ reported 11 cases of deep vein thrombosis (DVT) (incidence of 5.3%) when performing UGFS with up to 60ml of foam. European consensus guidelines recommend that foam volume should be limited to 10ml per session.^30^ We did not identify any severe adverse effects (AE). The most frequent AE was painful phlebitis in 4/22 patients (18.18%; 95%CI: 20.06-34.3%). 2/22 patients (9.09%; 95%CI: 0-21.10%) had venous path hardening and hyperemia without pain. In a randomized trial with patients with GSV reflux but without VU, Thomasset et al. identified superficial phlebitis in 18% of patients, pain in 14%, and skin staining in 28%. We observed skin staining in 3/22 (13.64%; 95%CI: 0-27.98%). Women had more adverse reactions than men, which is similar to the data reported by Thomasset et al.^31^ In a systematic review of 69 studies, Jia et al. reported the following frequent complications: 4.7% phlebitis, 17.8% skin staining, and 25.6% local pain.^32^ These complications rates are similar to those identified in the present study.

### Quality of life

We identified differences between men and women in terms of QoL improvement during the interval from 60 to 180 days after treatment. QoL is dependent on cultural factors and factors related to patients’ expectations. We believe residual varicose veins, incomplete VU healing, and occurrence of skin staining may be the causes of worse QoL in women compared to men at the end of the study. All of the patients who had VU that were not fully healed at 180 days after treatment were women. There was also a higher proportion of women in the group who had AE. Other outcomes in our study had similar results for both genders. Occurrence of residual varicose veins after treatment was not studied. The AQ aesthetic assessment would indicate whether women’s poor QoL was related to cosmetic factors.

### Clinical severity

Higher severity related to larger VU was to be expected, since they are scored higher on the VCSS. The influence of age is also compatible with the progressive nature of CVI. Scott et al. observed that patients with CVI were older than those with varicose veins without CVI.^33^ Large cross-sectional studies, point to a higher prevalence of varicose veins in women, but do not indicate a higher prevalence of severe CVI among women. Scott et al. identified that 60% of VU patients were men while there was a predominance of women among those with varicose veins. However, multivariate analysis revealed that CVI patients were older, male and obese. The small sample in our study may be responsible for inconsistent results and since in our study we recruited patients treated at the hospital, our sample may not reveal the actual prevalence but, rather, reflect the greater demand for treatment among women. In the Edinburgh study, the severe forms of CVI did not show a predilection for gender after adjustment for age.^34^

### Ulcer healing

We identified complete healing of 77.27% (CI 95% 59.76-94.78%) of VU and 90.90% (CI 95%78.89-100%) of VU diameters reduced during the study. Before treatment, the VU initially had dimensions ranging from 1 to 14cm (mean of 4.11cm and median of 3.00cm). Barwel et al. identified healing of 65% of VU after treatment by saphenectomy or compression.^12^ VU healing rates after UGFS range from 71 to 92%, but there are few randomized studies comparing sclerotherapy to clinical treatment and samples are also small.^22^^,^ 35 ^,^ 36 Campos Jr. et al. conducted a controlled study and demonstrated that UGFS and saphenectomy were similar, with healing of 91.3% of VU at 1-year follow-up.^22^ In a prospective cohort, Lloret et al. followed 180 UGFS-treated VU patients, 89 of whom (49.4%) had GSV reflux. At six years, 172 VU (95.6%) were healed. Lloret observed that deep venous reflux, VU open for more than 12 months, VU with an area of more than 6 cm^2^, lipodermatosclerosis, previous DVT, and bleeding were all associated with poorer cicatrization.^37^ Cabrera et al. also report worse results for healing of chronic VU and when there is deep venous reflux.^36^ The hypothesis that UGFS may be equivalent to surgery or thermoablation for averting recurrence of VU should be tested with long-term, controlled clinical trials with large groups. It is probable that USGFS is inferior for averting recurrence of VU, since foam sclerotherapy has higher rates of recanalization of treated veins and relapse of reflux.

### GSV Occlusion and elimination of reflux

We observed residual reflux in 7/22 (95%CI: 31.82% 12.36-51.28%) at 60 days and 8/22 (36.36%; 95%CI: 27.45-45.27) at 180 days. Total occlusion occurred in 10/22 (45.45%; 95%CI: 24.65-66.26%) at 60 days; and 7/22 (31.82%; 95%CI: 12.36-51.28%) at 180 days. GSVs with diameters from 5.5mm to 21mm (12mm +/-3.62) were treated. The large caliber may be related to low rates of occlusion and elimination of reflux. Jia et al. identified 87% occlusion of trunk veins treated with foam sclerotherapy.^32^ In a randomized trial, Brittenden et al. identified 54.6% occlusion of veins treated with sodium tetradecyl sulphate foam in a study that excluded GSVs with diameters exceeding 15 mm and in which only 2.8% of the patients had open or healed VU.^20^ We identified that GSVs that were completely occluded had smaller baseline diameters than GSVs that remained patent or partially patent, consistently, at both 60 days after treatment and at 180 days. Myers et al. prospectively studied 1189 sclerotherapy sessions performed in 489 patients with varicose veins, treating 454 GSVs. The occlusion rate after a single sclerotherapy session was 53.1%. The occlusion rate of non trunk veins was higher than for GSVs. Veins with a diameter greater than 6mm had worse results than those with diameters of 5mm or less. The best results were observed in patients older than 70 years when foam sclerotherapy was used and when foam volume greater than 12 ml and containing a higher concentration of sclerosants was used.^25^

## CONCLUSION

The benefits in terms of quality of life, ulcer healing, and reduction of the severity of the venous disease without serious complications suggest that sclerotherapy is a valid option.
